# Effect of coping materials zirconia or polyetheretherketone with different techniques of fabrication on vertical marginal gap and fracture resistance of posterior crowns with composite veneering

**DOI:** 10.1186/s12903-023-03247-w

**Published:** 2023-08-09

**Authors:** Marwa Emam, Mohamed F. Metwally

**Affiliations:** 1 Department of Fixed Prosthodontics, Faculty of Dentistry, Ain Shams University, Organization of African Unity St, El-Qobba Bridge, Al Waili, 11566 Cairo, Egypt; 2 Department of Crown and Bridge, Faculty of Dental Medicine, AL Azhar University, Cairo, Egypt

**Keywords:** Zirconia, Polyetheretherketone (PEEK), CAD/CAM, Pressing technology, Marginal adaptation, Fracture resistance

## Abstract

**Background:**

Insufficient research has been conducted in the literature assessing the performance of zirconia and polyetheretherketone (PEEK) crowns in relation to the essential requirements of successful restorations, such as fracture resistance or margin adaptation. The purpose of this study was to evaluate the effect of the coping materials zirconia or PEEK with different fabrication techniques on the vertical marginal gap and fracture resistance of posterior crowns with composite veneering.

**Methods:**

Ceramic copings (*n* = 18) restoring mandibular first molar were fabricated from zirconia (Zircon.x, Presidentdental, Germany), milled PEEK (PEEK CAD) (breCAM.BioHPP, Bredent, Germany) and pressed PEEK (PEEK Press) (BioHPP Granules, Bredent, Germany) six specimens each (*n* = 6). The copings were veneered with high impact polymer composite (HIPC) material (breCAM.HIPC, Bredent, Germany). The vertical marginal gap was captured under a magnification of 40X. Five equidistant marks on each surface of the die distinguished the points of measurement for a total of 20 readings per sample. The analysis was completed using an image analysis system (ImageJ 1.53t, National Institute of Health, USA). The specimens were loaded to failure at a crosshead speed of 1 mm/min and the load at failure was recorded to measure the fracture resistance.

**Results:**

The marginal gap was analyzed using one-way ANOVA followed by Tukey’s post hoc test. Fracture resistance was analyzed using Welch one-way ANOVA followed by the Games-Howell post hoc test. Marginal gap values showed a significant difference between the tested groups, with zirconia having significantly lower gap values (48.67 ± 11.98 µm) than both the PEEK CAD (108.00 ± 20.08 µm) and Press groups (108.00 ± 25.10 µm) (*p* < 0.001). However, the results of fracture resistance showed no significant difference (*p* = 0.06) with 1687.47 ± 253.29 N, 2156.82 ± 407.64 N, 2436.72 ± 725.93 N for zirconia, PEEK CAD, and Press, respectively. The significance level was *p* < 0.05.

**Conclusions:**

Zirconia framework crowns have a smaller vertical marginal gap than milled and pressed PEEK crowns. Crowns fabricated from zirconia, PEEK CAD, or PEEK Press frameworks and veneered with composite resin have comparable fracture resistance lower than the maximum biting force in the posterior region.

**Clinical relevance:**

Posterior crowns with zirconia frameworks are preferred over milled and pressed PEEK frameworks regarding margin adaptation, although all can safely survive the maximum occlusal forces without fracture.

## Introduction

Recently, developments in dental materials and technologies have given rise to a huge boost in the fabrication of indirect dental restorations [1, 2]. Computer-aided design and computer-aided manufacturing (CAD/CAM) technology allows for a superior outcome, more time efficiency, better predictability, and higher precision compared to conventional manufacturing techniques [3]. The success of dental restorations is dictated by three main factors: marginal fit, fracture resistance, and esthetics [4]. Zirconia is the most commonly used core material in all-ceramic prostheses due to its chemical stability, excellent biocompatibility, high compressive strength, and acceptable esthetics [5, 6]. On the other hand, the crystal structure in zirconia ceramics results in opacity. Therefore, zirconia must be coated with a suitable veneering material [7, 8]. Recently, translucent zirconia has been introduced with different coloring technologies, and it was recommended for clinical situations when a combination of high translucency and strength is needed. However, suboptimal esthetic properties are still a drawback [9]. Veneering of a zirconia framework results in even more esthetically acceptable restorations [10]. However, covering the zirconia core with porcelain veneering exposes the restoration to chipping or lamination of the veneer layer as one of the most frequent problems with zirconia restorations [11].

On the other hand, polyetheretherketone (PEEK) is a semicrystalline linear polycyclic aromatic polymer developed in 1978 [11]. PEEK shows high resistance to hydrolysis, chemical wear, and deterioration at high temperatures. Additionally, it offers superior mechanical properties, is biologically inert, and has a light density (1.32/cm^3^) and low modulus of elasticity (3–4 GPa) close to those of human bone [12–16]. All these attractive properties have favored the use of PEEK in dental applications. However, the usage of PEEK as full-cover monolithic restorations is constrained by aesthetic flaws, as low translucency and gray tint are among PEEK's optical characteristics. To achieve a satisfactory aesthetic result, a veneering composite layer is necessary [17].

Different methods can be used to process PEEK. One option is to vacuum-press the material in a dental technical laboratory. PEEK utilized for this purpose is either in granular form or industrially prepressed pellets. Another choice is milling utilizing CAD/CAM technology, in which milled PEEK blanks are industrially pressed using predetermined conditions such as pressure, temperature, and time [18].

Excellent optical and mechanical qualities have been made achievable by improvements in adhesive technology and newer composite resins. Composite resin veneering can be used to support and enhance ceramic crowns [19]. In regard to cost, accuracy, and conservation, repairing composites is far simpler than repairing ceramics or replacing the whole restoration [20]. Moreover, veneering using composite resin could prevent enamel abrasion caused by the ceramic veneer layer [21].

Marginal adaptation is crucial in determining how well a restoration performs over the long run. To prevent wear of the luting cement, a marginal adaptation value in clinical situations for ceramic restorations of up to 120 µm was considered acceptable [22]. Poor marginal adaptation can cause dental plaque buildup, secondary caries, periodontal disorders, and eventually tooth loss [23]. Assessing the marginal adaptation of ceramic restorations utilizing both destructive and nondestructive methods has been described in previous studies [24–28]. While nondestructive methods include silicone replica with stereomicroscopy, resin replica with scanning electron microscopy (SEM), microcomputed tomography (CT), and optical coherence tomography (OCT), destructive methods include cutting samples into slices and measuring with a stereomicroscope [24]. According to Nawafleh et al. [26], the most popular method for obtaining reliable results was the direct view technique of marginal adaptation of the restorations under scanning electron microscopy (SEM), digital microscopes or stereomicroscopes.

Moreover, the most frequent cause for replacing dental prostheses is fracture. Therefore, it is crucial to assess a dental material's fracture resistance before employing it as a long-term permanent restoration in different clinical conditions [29].

The use of zirconia, pressed and milled PEEK crowns has been established in fixed prosthodontics. However, the performance of crowns regarding fundamental criteria of successful restorations, such as margin adaptation or fracture resistance, has not been adequately investigated in the literature. Therefore, this study aimed to assess the effect of the coping materials zirconia or PEEK with different techniques of fabrication, PEEK CAD or press, on the vertical marginal gap and fracture resistance of posterior crowns with composite veneering. The null hypothesis was that neither the material type nor the PEEK fabrication technique affects the restoration’s marginal adaptation or fracture resistance.

## Materials and methods

**Fig. 1:**
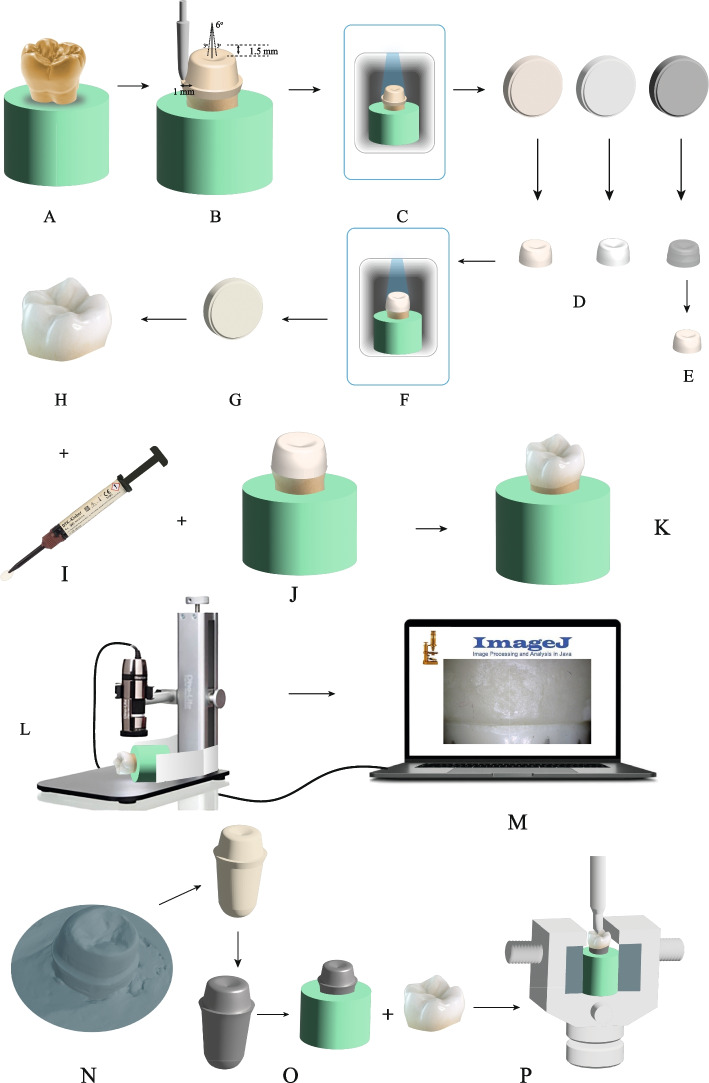
An overview of the study design

A power analysis test was conducted (G* Power v 3.1.9.2, Heinrich–Heine–Universität, Düsseldorf, Germany) based on the results of a previous study [23], and a sample size of 3 in each group had 95% power, an effect size of 4.26 with a significance level alpha (α) of 0.05 (two-tailed). A total of 18 crowns (*n* = 18) were considered adequate in this study and divided into three groups according to the type of material and technique of fabrication of the PEEK coping; zirconia, milled PEEK CAD, and PEEK press six samples each (*n* = 6). A study overview is shown in Fig. 1. (Study overview: A: A typodont molar was fixed in an acrylic base. B: The molar was prepared with the specified dimensions. C: The preparation was scanned to design a veneered crown. D: Copings were milled out of PEEK CAD, zirconia and wax. E: Wax copings were invested and PEEK Press copings were fabricated. F: Each coping was separately scanned for a veneering. G: HIPC composite blocks were milled. H: manufacturing HIPC veneering. I & J: Bonding veneering with DTK-Kleber adhesive cement to corresponding copings. K: Each sample was seated on the prepared molar and used for margin measurement. L: A digital microscope was employed for vertical gap measurements after fixing the samples with a custom holder. M: ImageJ software was utilized for margin image analysis. N: The STL file of the prepared molar was used for printing a resin die. O: The resin die was cast to a metal die that was fixed in an acrylic base. P: The samples were loaded to failure, and fracture resistance values were recorded).

### Preparation of specimens

A mandibular first molar typodont tooth was fixed with the aid of a parallelometer (BEGO. PARASKOP, Germany) in a self-cured acrylic resin base (Acrostone, Egypt) in an upright position. The top surface of the acrylic resin was 2 mm apical to the cervical line of the tooth. After the complete setting of the base, the tooth was prepared to receive an all-ceramic crown. The criteria of the preparation were a 1 mm deep chamfer finish line and a 1.5 mm occlusal clearance. A laboratory diamond abrasive bur with a round tip and 6° taper angle attached to a milling surveyor (BEGO. PARASKOP, Germany) was used for the axial preparation. A digital impression was produced by scanning the prepared tooth after spraying it with titanium dioxide powder using a dental lab scanner (Freedom HD, DOF, Inc., Seoul, South Korea). Full anatomical crowns were designed using designing software (Exocad Dental CAD; exocad GmbH, Germany) to have axial and occlusal dimensions of 1.5 mm followed by virtual reduction to attain 0.5 mm minimum thickness of the coping framework using digital cutback technique. The spacer thickness for cement was set at 50 µm starting 1 mm from the finish line margin. The designed files were imported to the 5-axis milling machine (Machine DWX-51D Dental Milling Machine; Roland DG, Frenchs Forest, Australia) for milling of six zirconia (Zircon.x, Presidentdental, Germany), six PEEK copings (breCAM. BioHPP, Bredent, Germany), and six wax patterns to be later employed for manufacturing the PEEK pressed group. The zirconia copings were then sintered according to the manufacturer’s instructions at 1530^○^C for 2 h in a sintering furnace (Nabertherm, Germany). The milled wax patterns were transformed into PEEK by lost wax and heat pressing procedures. The wax patterns were sprued and invested with phosphate-bonded investment material (Brevest For2press, Bredent, Germany). After preheating, PEEK granules (BioHPP Granules, Bredent, Germany) were pressed using a press device (For2press, GmbH & Co KG, senden, Germany). Crowns were divested after cooling with the aid of a blasting device (Basic Classic, Renfert GmbH, Germany) utilizing 110 μm alumina particles at 3 bar pressure before finishing and polishing following the manufacturer’s instructions combining a rubber polisher with polishing paste.

### Construction and cementation of the composite veneering

**Fig. 2:**
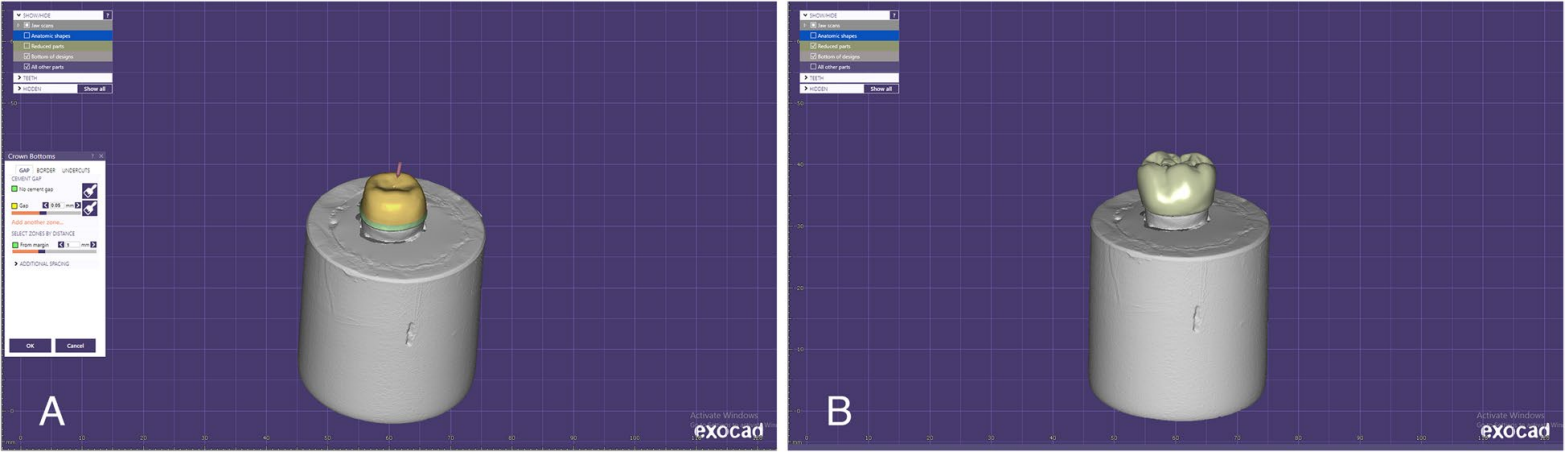
The copings (**a**) and veneering composite (**b**) design in Exocad designing software

The frameworks were individually scanned and superimposed with the full anatomical design to subtract the veneering parts that were later milled in high impact polymer composite (HIPC) (breCAM.HIPC, Bredent, Germany), and a spacer of 50 µm was assigned for cement space between the framework and veneering (Fig. 2). All frameworks and veneers were air abraded at the bonding surfaces with 110 μm Al_2_O_3_ powder at 2.5 bar and 3 cm distance. The HIPC composite veneers for all groups and PEEK frameworks were conditioned using PMMA and composite primer (Visio.link, Bredent, Germany) and light cured for 90 s using bre lux Power light curing Unit 2 (bredent, Senden, Germany) (intensity: 220 mW/cm2) at a wavelength between 370–500 nm for 90 s. Zirconia frameworks were conditioned after sandblasting using metal and ceramic primer (MKZ, Bredent, Germany) and cured for 90 s. Veneers were cemented to frameworks with dual-cure composite adhesive cement (DTK-Kleber adhesive, Bredent, Germany) and cured for 180 s. Excess material was removed, and samples were finished and polished using a Visio.lign finishing and polishing tool kit (Bredent, Gmbh, Germany).

### Vertical marginal gap distance measurements

**Fig. 3:**
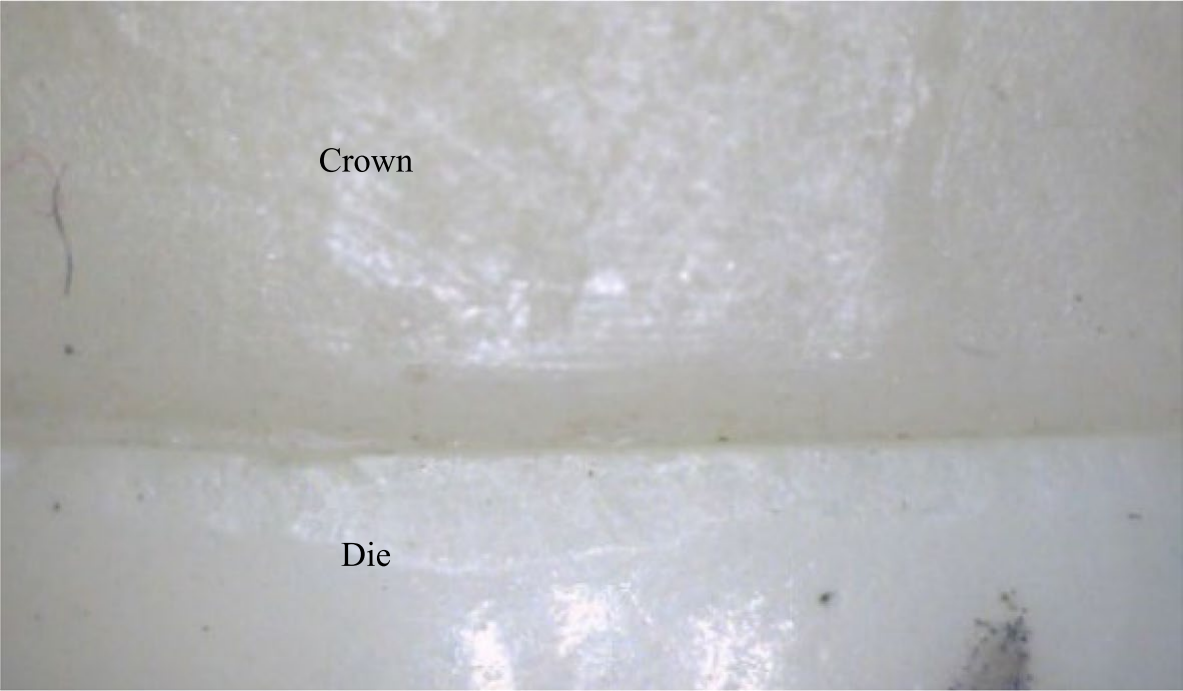
A photo of a zirconia sample ready for margin measurements with ImageJ analysis software

The vertical marginal gap was captured using a hand-held digital microscope with a built-in camera fitted on a precision microscopic stand (Dino-Lite Pro, Olympus, Tokyo, Japan) and connected to a personal computer using a magnification of 40X. Before initiating the measurements, the microscope's calibration instructions were accurately performed. The vertical gap was calculated as the distance between the finishing line's outermost margin and the restoration's most exterior cervical edge [30] at the same marked points in the four axial surfaces of all groups. Specimens were held in place over the die with a custom holding device, and five equidistant marks on each surface of the die distinguished the points of measurement for a total of 20 readings per sample before averaging them into one value. The analysis was completed using open-source software for processing and analyzing scientific images (ImageJ 1.53t, National Institute of Health, USA) (Fig. 3).

All measurements and the analysis of all photos were conducted by the same researcher (M.E) for standardization purposes.

### Fracture resistance measurements

**Fig. 4:**
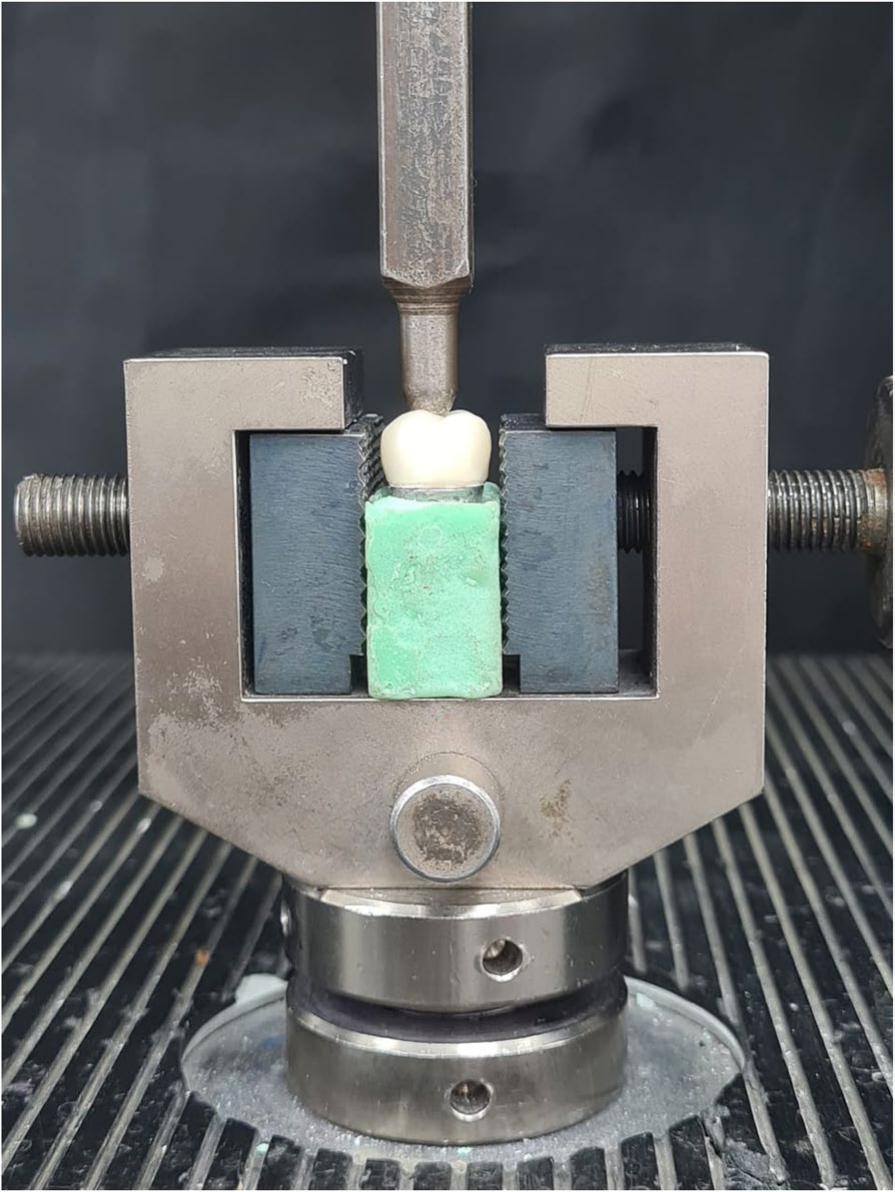
Fracture load testing

The STL file of the digital impression captured previously for the prepared abutment was used to print a resin (Savoy C&B resin, China) abutment with the exact same dimensions using a 3D printer (Photon S, Anycubic, China). Later and after post curing, the resin die was invested and cast to fabricate a cobalt-chromium metal tooth model. The metal die was fixed in a self-cured acrylic resin base (Acrostone, Egypt) in an upright position. The crowns were checked on the die to ensure that they had the exact same fit as on the typodont prepared abutment. In the universal testing machine, each specimen from the three tested groups was placed individually on the metal die and held in place by the holder in the lower compartment of the machine before being loaded compressively at a crosshead speed of 1 mm/min. A vertically movable rod with a 5 mm-diameter semispherical head was positioned directly over the occlusal surface in the central fossa to ensure a uniform distribution of stresses. The value associated with the first break in the loaded specimen served as the failure load or fracture resistance value. As soon as the load dropped by 30% from the maximum load, the load at failure was confirmed and recorded in Newtons (Fig. 4).

### Statistical analysis

The data obtained from both vertical marginal gap distance measurements and fracture resistance testing were collected, tabulated, and then subjected to statistical analysis.

Numerical data are presented as the mean, standard deviation (SD), median and interquartile range (IQR). Shapiro‒Wilk's test was used to test for normality. The homogeneity of variances was tested using Levene's test. Data were normally distributed, but the homogeneity assumption was violated in fracture resistance data, so they were analyzed using Welch one-way ANOVA followed by the Games-Howell post hoc test. Marginal gap values were analyzed using one-way ANOVA followed by Tukey’s post hoc test. The significance level was set at *p* < 0.05 within all tests. Statistical analysis was performed with R statistical analysis software version 4.2.3 for Windows [31].

## Results

**Fig. 5:**
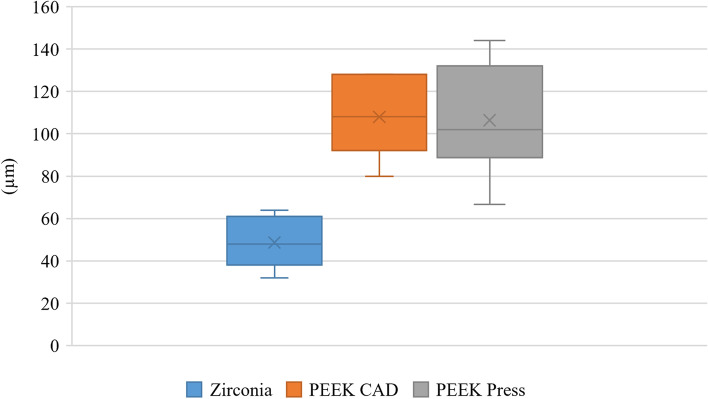
Box plot showing marginal gap (µm) values in different groups

**Fig. 6:**
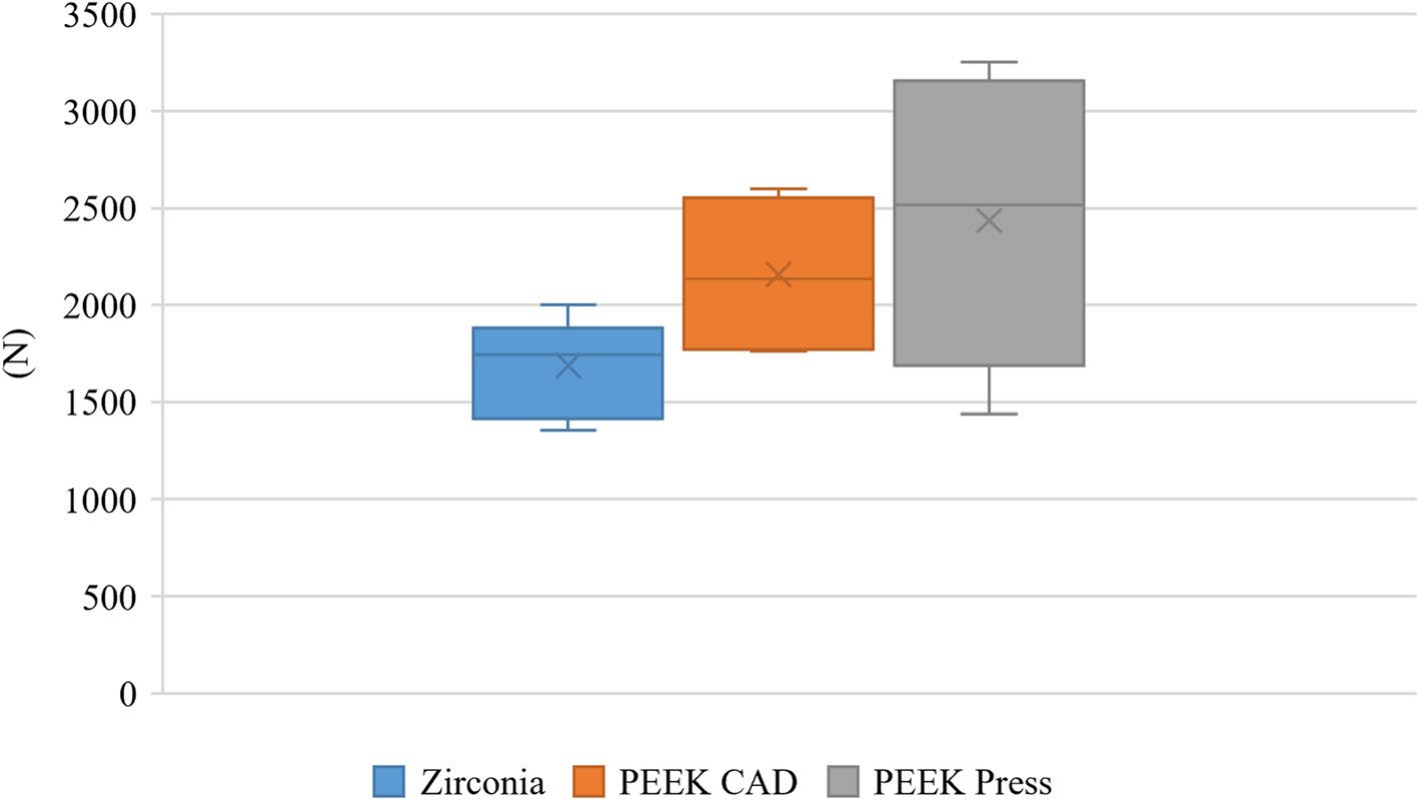
Box plot showing fracture resistance (N) values in different groups

Descriptive statistics for marginal gap values and fracture resistance are presented in Table 1 and Figs. 5 and 6 respectively. The results of the intergroup comparison of marginal gap values presented in Table 2 showed that there was a significant difference between the tested groups, with zirconia samples having significantly lower gap values than the PEEK groups (*p* < 0.001). However, the results of intergroup comparisons of fracture resistance values presented in Table 3 showed that there was no significant difference between the tested groups (*p* = 0.06).

**Table 1:** Descriptive statistics

Measurement	Group	Mean	95% CI		SD	Median	IQR
			**Lower**	**Upper**			
**Marginal gap (µm)**	**Zirconia**	48.67	39.08	58.25	11.98	48.00	15.00
	**PEEK CAD**	108.00	91.93	124.07	20.08	108.00	30.00
	**PEEK pressed**	108.00	89.41	126.59	25.10	108.00	26.66
**Fracture resistance (N)**	**Zirconia**	1687.47	1484.80	1890.14	253.29	1743.50	355.01
	**PEEK CAD**	2156.82	1830.64	2482.99	407.64	2134.65	725.45
	**PEEK pressed**	2436.72	1855.86	3017.57	725.93	2517.95	1091.95

*95% CI* 95% confidence interval for the mean, *SD* Standard deviation, *IQR* Interquartile range

**Table 2:** Intergroup comparisons and summary statistics of marginal gap values (µm)

Marginal gap (µm) (Mean ± SD)			f value	*p* value
**Zirconia**	**PEEK CAD**	**PEEK Press**		
48.67 ± 11.98^B^	108.00 ± 20.08^A^	108.00 ± 25.10^A^	**17.75**	< 0.001*

Means with different superscript letters within the same horizontal row are significantly different *significant (*p* < 0.05)

**Table 3:** Intergroup comparisons and summary statistics of fracture resistance values (N)

Fracture resistance (N) (Mean ± SD)			f value	*p* value
**Zirconia**	**PEEK CAD**	**PEEK Press**		
1687.47 ± 253.29^A^	2156.82 ± 407.64^A^	2436.72 ± 725.93^A^	**3.41**	**0.06**

Means with different superscript letters within the same horizontal row are significantly different *significant (*p* < 0.05)

**Fig. 7:**
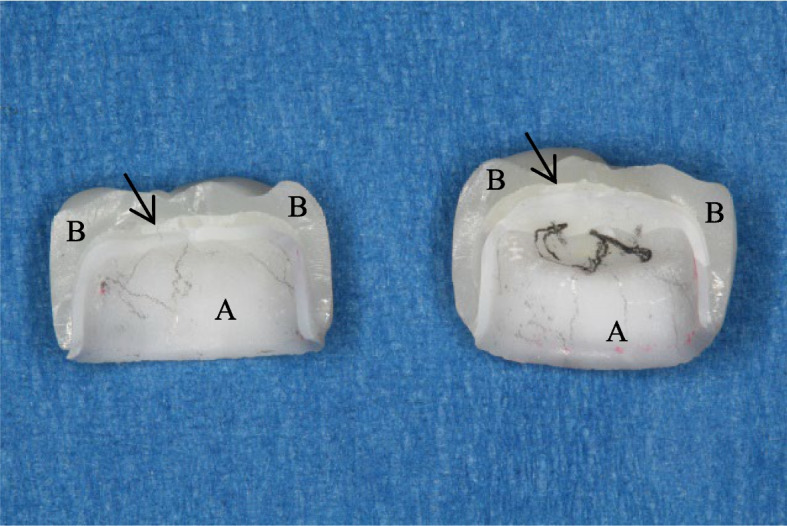
A fractured zirconia crown. **A** Coping, **B** Veneering, arrows point to the cement layer

**Fig. 8:**
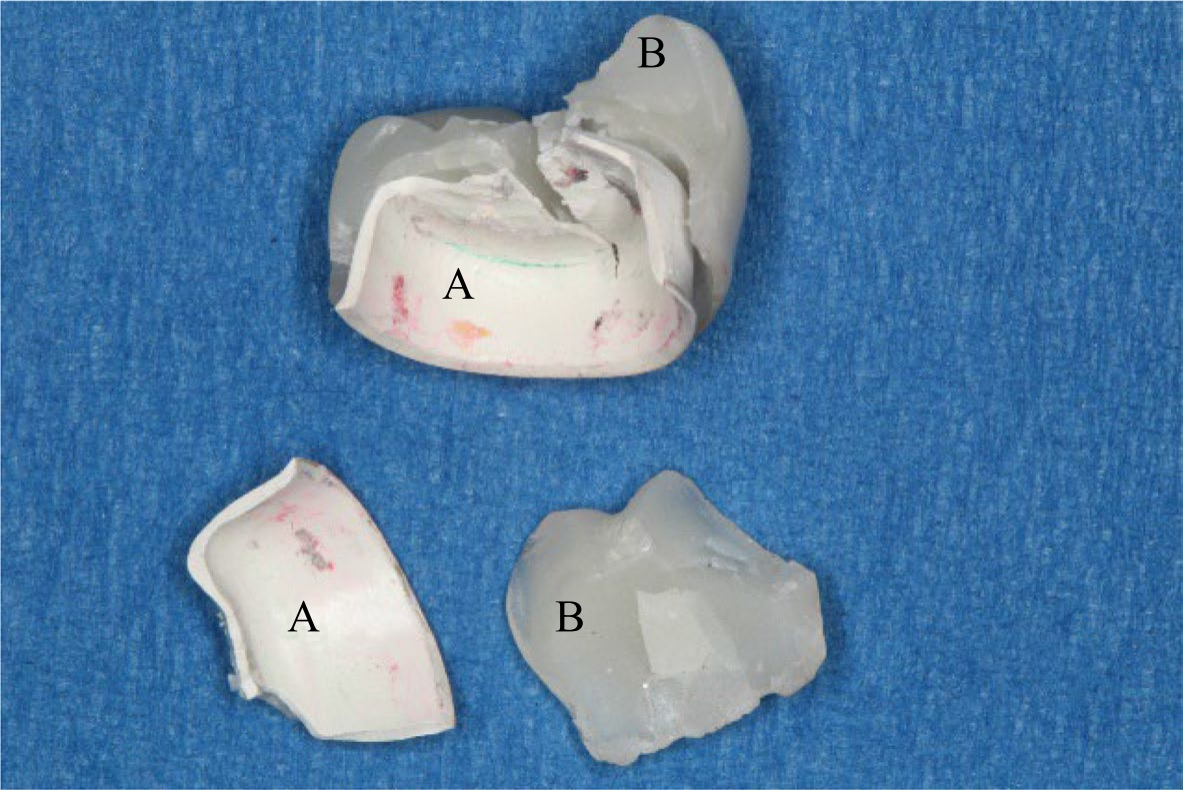
A fractured PEEK CAD crown. **A** Coping, **B** Veneering

**Fig. 9:**
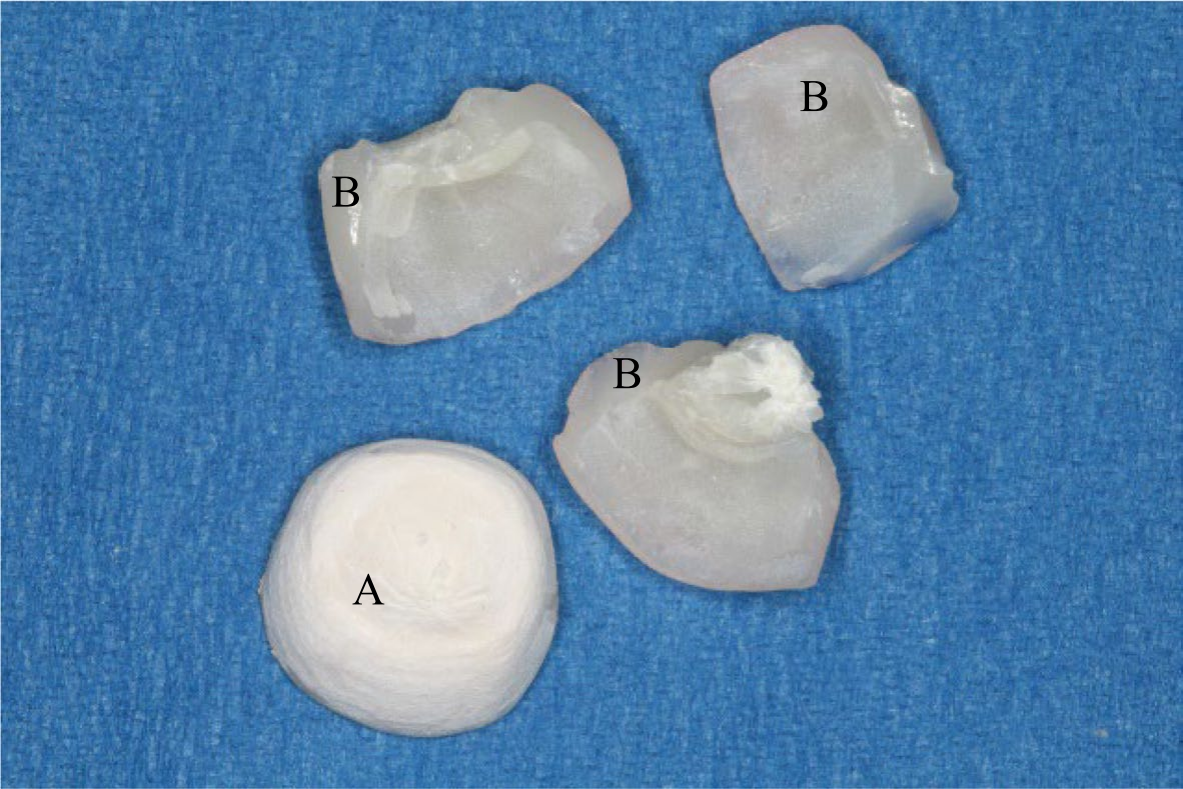
A fractured PEEK Press crown **A** Coping, **B** Veneering with attached resin cement to fitting surface

Regarding the mode of failure analysis, all zirconia and PEEK CAD samples showed catastrophic fracture of the coping and veneering (Figs. 7 and 8), while all PEEK Press samples failed with intact coping, fractured, and separated veneering (Fig. 9) and Table 4.

**Table 4:** The percentage of the failure mode in each group

Group	Catastrophic fracture	Fracture and separation of veneering
**Zirconia**	100%	0%
**PEEK CAD**	100%	0%
**PEEK Press**	0%	100%

## Discussion

The null hypothesis was partially accepted, as there was no significant difference between the fracture resistance of crowns fabricated from the tested framework materials. However, there was a significant difference between the tested groups regarding marginal gap distance, with zirconia samples having significantly lower gap values than the PEEK CAD and press groups.

All this study’s vertical marginal gap values, however, fell within acceptable limits. According to the authors, vertical marginal gaps for fixed dental prostheses (FDPs) < 120 µm were considered clinically acceptable [32]. Others have stated that clinical acceptance for conventional crowns ranges from 160 to 172 µm [33, 34].

To avoid the effects of uncontrolled finger pressure or overfilling the crown with cement, which may result in an uneven flow of cement with some axial walls having a thick layer and the opposite walls having a thinner layer, the assessment of marginal discrepancy was carried out in our study before cementation [26]. For in vitro testing, a minimum established requirement of 20 measurements per specimen was considered essential [35].

Direct microscopic examination was implemented in this study as it was considered less time-consuming than other methods and less likely to lead to the accumulation of errors that may arise from many steps and ultimately affect the accuracy of results [26].

In the present study, the mean marginal gap values were 48.67 ± 11.98 µm for the zirconia group, 108.00 ± 20.08 µm for the PEEK CAD group, and 108.00 ± 25.10 µm for the PEEK Press group. As fully sintered zirconia demonstrated marginal discrepancy values ranging between 60.4 and 110.1 µm, while partially sintered zirconia showed marginal discrepancy values between 24.6 and 65 µm, the results of this study were considered within the range established by previous studies [36–38].

Zeighamie et al. [39] compared the marginal adaptation of implant-supported frameworks made of PEEK, zirconia, and composite. They ultimately concluded that zirconia frameworks exhibited greater marginal adaptation (33.25 ± 26.51 µm) than PEEK (92.40 ± 40.00 µm) and composite (63.17 ± 46.02 µm) frameworks.

Baran et al. [23] compared the marginal and internal adaptation of three-unit FDPs fabricated from cubic zirconia, fiber-reinforced resin composite, PEEK, polyetherketoneketone (PEKK), and polymer composite material using the silicone replica technique at 40X magnification under a stereomicroscope. The marginal gap values for the cubic zirconia material (51.76 ± 7.31 μm) were found to be significantly lower than those seen in the other materials with 67.44 ± 5.52 μm for the PEEK group, although all materials showed measurements within the clinically acceptable range.

Meshreky et al. [40] evaluated the vertical marginal gap of PEEK veneered with milled HIPC compared to zirconia veneered with CAD-On lithium disilicate glass ceramic. They concluded that PEEK crowns had a greater marginal gap (49.88 ± 7.97 µm) than zirconia crowns (18.39 ± 3.1 µm). Roy et al. [41] reported the same conclusions in an in vivo experiment. Moreover, Aula RA et al. [42] concluded that PEEK crowns had a marginal gap of 409.09 μm, 112.86 μm, and 198.56 μm for proximal, buccal and lingual surfaces, respectively, which is higher than the clinically acceptable limit, while zirconia crowns exhibited much lower measurements, 105.08 μm proximal, 27.65 μm buccal, and 45.13 μm lingual. The difference might be attributed to the variation in methodology, as they used 3-unit FDP, and the marginal gap was assessed using a stereomicroscope at 160X.

The degree of a material's stiffness and its capacity for internal and marginal adaptability have been found to be positively correlated. The zirconia material has a bending strength of 1250 MPa, whereas high-performance polymers are known to have bending values equivalent to those of dentin. In light of these results, the zirconia group's improved marginal and internal adaptation in the current investigation can be attributed to the material's enhanced stability during milling compared to high-performance polymers [23].

Moreover, these findings might be due to PEEK's semicrystalline structure, which includes fillers entrapped in a resin matrix yielding a greater marginal gap during manufacturing than zirconia, which has a polycrystalline structure [42].

Our study results are not in agreement with Park JY et al. [43] and Hossam et al. [44], who found no significant difference between margin gap measurements of zirconia and PEEK crowns. Amalorpavam et al. [45] found less marginal fit (50.26 ± 16.02 µm) and internal adaptation (32.8 ± 5.2 µm) in zirconia copings when compared to the PEEK copings (30.3 ± 5.1 µm) for marginal gap, (29.1 ± 5.8 µm) for internal gap and the difference was statistically significant. This was attributed to the shrinkage occurring in the zirconia framework after sintering. The variation among results was attributed to different methodological steps, as in the study conducted by Amalorpavam et al. [45] The samples were sectioned and scanned under a field emission scanning electron microscope for marginal fit. In addition, only two points per sample were selected to assess the marginal gap distances.

Mostafa et al. [46] evaluated the effect of fabrication techniques on the marginal and internal adaptation of PEEK molar single crowns. It was concluded that PEEK CAD crowns demonstrated higher marginal accuracy and nearly similar internal fit when compared to PEEK pressed crowns.

Additionally, the impact of various manufacturing methods on the marginal precision of PEEK single-crown copings was examined by Attia et al. [47]. The average marginal gap was reported to be 72 ± 9 µm for the PEEK Press pellet group and 45 ± 6 µm for the PEEK CAD/CAM group.

The peripheral and internal fit of copings made of PEEK and zirconia were assessed by Beuer et al. [48] There was no significant difference between the three groups. Whereas the PEEK-CAD result was 130 ± 40 µm, the PEEK-pressed result was 112 ± 40 µm.

Makky et al. [49] assessed the marginal and internal fit of pressable versus machinable PEEK and versus zirconia copings. It was established that the marginal and internal fit for the three groups were within the acceptance range, while zirconia copings showed significantly superior marginal fit compared to the PEEK groups. The PEEK CAD showed mean vertical marginal measurements of 130 ± 40 µm, while the PEEK press showed 112 ± 40 µm.

Sokkary et al. [50] assessed restoration fracture, margin adaptation, and patient satisfaction to compare the clinical performance of single crowns fabricated of zirconia and milled PEEK after one year. Regarding mechanical aspects, marginal integrity and patient satisfaction, there was no significant difference between the two materials.

In one study [41], stereomicroscopy revealed a higher marginal gap of PEEK crowns, whereas cone beam computed tomography (CBCT) revealed the contrary, with porcelain fused to metal (PFM) having a much larger marginal gap. From this, it could be concluded that the method of detecting the marginal gap affects the measured values.

Regarding fracture resistance, there was no significant difference between the fracture resistance of crowns fabricated from zirconia, PEEK CAD and press groups, with all showing higher fracture load than the maximum biting force (800 – 1000 N) [51]. The optimum modulus of elasticity reported for PEEK material is closer to composite material and dentin, which may diminish stress induction at the interface layer at different layers of the crown and account for the higher yet nonsignificant fracture strength values for PEEK veneered crowns [50].

The results could also be attributed to the physical and chemical structure dissimilarity of zirconia and veneering composite compared to PEEK substructure veneered with a similarly based polymer composite veneering [21].

These findings were consistent with a previous study concluding that the composite veneered PEEK crown group recorded higher fracture resistance values at 1327.18 ± 44.03 N, followed by zirconia veneered with composite crowns (1196.94 ± 52.10. N), which is consistent with the results of the current study [21]. Another study [52] investigated the fracture resistance of CAD/CAM implant abutments fabricated of titanium, zirconia, and PEEK and supporting crowns of lithium disilicate ceramic, and no significant difference was found between zirconia (623.9 ± 97.4 N) and PEEK (602.9 ± 121 N).

On the other hand, our study results disagree with those of Tartuk et al. [53], who compared the load-bearing capacities of PEEK, hybrid ceramic and zirconia crowns manufactured using CAD/CAM. There was no statistically significant difference between the PEEK (2214 ± 236 N) and hybrid ceramic groups (2325 ± 264 N); nevertheless, the zirconia group had the highest values regarding fracture load (3292 ± 192 N). They used a zirconia die and monolithic restorations instead of the metal die and composite veneering in our case. Stawarczyk et al. [18] examined the failure loads of three PEEK FDPs made utilizing different fabrication methods. The mean fracture load of PEEK CAD (2354 N) was higher than that of PEEK pressed from granular material (1738 N). Lack of veneering and different restoration designs might contribute to the variation in results, as the stress concentrates in the connector area in FDP, which was not the case in this study.

Regarding the mode of failure analysis, in a previous study [18], FDPs manufactured from prepressed CAD/CAM blocks and FDPs pressed from pellet PEEK both showed complete fractures at the pontic; however, FDPs made from granular PEEK generally showed plastic deformation with an assumed loss in elastic deformation properties. These results are in agreement with the failure modes noticed in this study, as PEEK was pressed from granules rather than pellets, which explains the intact copings that might have deformed, causing separation and fracture of the veneering composite in this group.

The validity of comparing the results of the present study and those obtained in prior investigations is hindered by factors including the wide range of manufacturing techniques, the use of different materials and designs of master models, different numbers of restoration units, number of sample sizes, the study design (in vitro or in vivo), different measurement sites for margin measurements, different die materials used in fracture testing and the use of different cement thickness values [23, 46].

Future studies with aging by chewing simulation or thermocycling are needed since specimens in this study were not aged and considered among the limitations. Furthermore, clinical research is also required to back up the application of PEEK for long-term treatments.

## Conclusions

1. Zirconia framework crowns have less vertical marginal gap than PEEK regardless of its fabrication technique, with all falling within the clinically acceptable range of < 120 µm.
2. The technique of fabrication of PEEK with milling or pressing did not affect the vertical marginal gap of posterior crowns.
3. Crowns fabricated from zirconia, PEEK CAD, or PEEK Press frameworks and veneered with composite resin have comparable fracture resistance lower than the maximum biting force in the posterior region.
4. Composite veneered crowns fabricated from zirconia and PEEK CAD fail by complete fracture of the coping and veneering, while PEEK Press copings survive the applied load and show fracture and separation of the veneering composite.

## Data Availability

Datasets analyzed during the current study are available from the corresponding author on reasonable request.
